# Impact of Exposure Duration to High-Altitude Hypoxia on Oxidative Homeostasis in Rat Brain Regions

**DOI:** 10.3390/ijms26178714

**Published:** 2025-09-07

**Authors:** Boris Lira-Mejía, Roger Calderon-Romero, Jorge Ordaya-Fierro, Cristian Medina, José-Luis Rodríguez, Alejandro Romero, Roberto Dávila, Mariella Ramos-Gonzalez

**Affiliations:** 1 GIFATA Research Group, Animal Physiology Laboratory, Faculty of Veterinary Medicine, Major National University of San Marcos, Lima 15021, Peru; roger.calderon@unmsm.edu.pe (R.C.-R.);; 2 Complutox Research Group, Department of Pharmacology and Toxicology, Faculty of Veterinary, Complutense University of Madrid, 28040 Madrid, Spain; manarome@ucm.es; 3 Veterinary Clinic, Faculty of Veterinary Medicine, Major National University of San Marcos, Lima 15021, Peru; 4 Zootecnia an Animal Production Laboratory, Faculty of Veterinary Medicine, Major National University of San Marcos, Lima 15021, Peru; mramosgo@unmsm.edu.pe

**Keywords:** high altitude, hypoxia, brain regions, antioxidant enzymes, exposure duration, oxidative stress, neuroinflammation

## Abstract

Hypoxia at altitudes above 3000 m poses a significant threat to organ health and physiological homeostasis, particularly in metabolically active tissues such as the brain. Many of the cellular alterations induced by hypoxia are associated with the excessive generation of reactive oxygen species (ROS) and the resulting oxidative stress. In this study, we investigated the effects of exposure duration and altitude levels on oxidative homeostasis in the rat hypothalamus, cortex, hippocampus, and striatum. We assessed ROS production, malondialdehyde (MDA) levels, the antioxidant activities of superoxide dismutase (SOD), and catalase, as well as molecular biomarkers of oxidative stress, cell death, and inflammation. Our findings demonstrated that ROS, MDA and SOD levels increased across all brain regions, particularly in response to higher altitude exposure. Conversely, catalase activity decreased under the same conditions. At the molecular level, we observed overexpression of key biomarkers related to oxidative stress, cell death, and inflammation, especially at extreme altitudes. Furthermore, these effects were most pronounced in the hippocampus, cortex, and striatum. In conclusion, our data indicate that hypoxic exposure at higher altitudes significantly contributes to the oxidative disruption of brain homeostasis in rats.

## 1. Introduction

Oxygen is essential for maintaining cellular homeostasis and organ function, particularly in metabolically demanding tissues such as the brain. Due to its high energy requirements and limited capacity for anaerobic metabolism, the brain is especially vulnerable to hypoxic conditions, which can rapidly impair neuronal integrity and function [1].

Hypoxia, defined as a deficiency in the amount of oxygen reaching the tissues, is a common physiological stressor at high altitudes, typically above 3000 m (PO_2_: 110.2 mmHg), where the partial pressure of oxygen significantly decreases [2]. This drop in oxygen availability triggers a cascade of physiological and molecular adaptations aimed at preserving oxygen delivery and cellular function [3,4]. However, these compensatory mechanisms are often insufficient to fully counteract the harmful effects of oxygen deprivation, particularly during acute or prolonged exposure [5,6].

High-altitude hypoxia poses a serious threat to systemic homeostasis, with the brain being among the most susceptible organs [6]. The pathophysiological effects of hypoxia in the brain are multifactorial, involving alterations in cerebral blood flow, disruptions in energy metabolism, and, most critically, the induction of oxidative stress [3]. Oxidative stress arises when the generation of reactive oxygen species (ROS) exceeds the capacity of endogenous antioxidant defenses, resulting in damage to cellular macromolecules and the activation of pro-inflammatory and pro-apoptotic signaling pathways [3,5,6].

A growing body of evidence indicates that high-altitude exposure increases ROS generation and promotes the accumulation of oxidative stress biomarkers in the brain [3,5,6,7]. These alterations have been observed across several brain regions, including the hypothalamus, cortex, hippocampus, and striatum [4,6,7]. A hallmark of a hypoxia-induced brain injury is the imbalance between ROS production and antioxidant enzyme activity, particularly involving superoxide dismutase (SOD) and catalase [6]. Elevated levels of malondialdehyde (MDA), a biomarker of lipid peroxidation, further reflect the extent of oxidative damage under hypoxic conditions [6,8]. Additionally, hypoxia-induced oxidative stress is often associated with the overexpression of genes involved in inflammation and apoptosis, contributing to neuronal vulnerability and the pathogenesis of acute and chronic neurological disorders [3,9].

The molecular mechanisms underlying hypoxia-induced neuronal injury include both necrotic and apoptotic pathways, with the balance of pro- and anti-apoptotic gene expressions influencing cell fate [9,10]. Hypoxia activates intracellular signaling pathways such as the mitogen-activated protein kinase (MAPK) cascade, which modulates antioxidant defense, metabolism, and cell death [10]. Hypoxia-inducible factor 1α (HIF-1α) also plays a central role in the cellular adaptation to oxygen deprivation by regulating genes involved in metabolic reprogramming [11], inflammation [12], and survival [13]. Moreover, neuroinflammation, driven by microglial activation and the release of pro-inflammatory cytokines such as TNF-α and IL-1β, can further aggravate neuronal damage and disrupt the blood–brain barrier (BBB) under hypoxic conditions [1,14].

Environmental hypoxia promotes the activation of the NF-κB pathway and induces expression of key inflammatory genes, including TNF-α, IL-6, and COX-2. Specifically, hypoxic exposure significantly increases COX-2 expression, both in vivo and in cell cultures systems. Under hypoxic conditions, cells exhibit slow but sustained NF-κB activation through IκBα degradation, subsequently inducing IL-6 transcription. This activation process is ROS-dependent, as evidenced by its inhibition in the presence of antioxidants. Moreover, studies using human endothelial cells have confirmed that hypoxia-induced COX-2 expression is directly mediated by NF-κB p65, which binds to the COX-2 promoter region, thereby reinforcing the central role of this pathway in the orchestrating inflammatory responses. Additionally, TNF-α has been documented as a potent NF-κB activator, creating a positive feedback loop that amplifies the inflammatory cascade. Collectively, these molecular events constitute an adaptive inflammatory response to environmental hypoxia, mediated by an NF-κB-centered regulatory network that coordinates the expression of pro-inflammatory cytokines and enzymes [15,16,17].

Hypoxic conditions can induce overexpression of BAX, a proapoptotic member of the Bcl-2 protein family that facilitates outer mitochondrial membrane permeabilization and subsequent cytochrome c release into the cytosol [18]. Released cytochrome c interacts with APAF-1 (apoptotic protease activating factor 1) and ATP to assemble the apoptosome, a multiprotein complex that recruits and activates procaspase-9. Activated caspase-9 subsequently cleaves and activates caspase-3, a critical executioner caspase that degrades essential structural and regulatory cellular substrates [19]. Hypoxic stress also upregulates BNIP3 expression, a proapoptotic and mitophagic protein under HIF-1 transcriptional control that disrupts anti-apoptotic Bcl-2/Bcl-xL interactions and contributes to mitochondrial membrane potential dissipation [20]. Therefore, environmental hypoxia integrates mitochondrial and cytosolic signaling pathways to coordinate activation of BAX, BNIP3, APAF-1, and caspase-3 within a functional apoptotic axis that drives programmed cell death and tissue remodeling under sustained hypoxic stress conditions.

Despite increasing recognition of oxidative stress as a key mediator of hypoxia-induced brain injury, the specific contributions of altitude level and exposure duration to these molecular events remain poorly understood [21]. While previous studies suggest that both factors influence oxidative responses, comprehensive evaluations across distinct brain regions are still limited [6]. Furthermore, the regional specificity of antioxidant defenses and the interaction between oxidative and inflammatory pathways require further investigation to identify effective targets for neuroprotection [6,8,10].

This study aims to address these gaps by systematically analyzing the effects of exposure duration and altitude level on oxidative stress markers in key brain regions of a rat model. We evaluated ROS production, MDA levels, the enzymatic activities of SOD and catalase, and the expression of molecular markers related to oxidative stress, inflammation, and apoptosis in the hypothalamus, cortex, hippocampus, and striatum. By characterizing the spatial and temporal dynamics of oxidative responses under hypoxic conditions, this work aims to advance our understanding of high-altitude-induced brain injury and to inform future neuroprotective strategies.

In summary, our findings provide new insights into the disruption of redox homeostasis in the brain under extreme altitude exposure. The results underscore the critical role of altitude level in driving oxidative damage and highlight potential molecular targets for mitigating hypoxia-related neurological impairments.

## 2. Results

### 2.1. Antioxidant Function and Oxidative Stress

**Figure 1:**
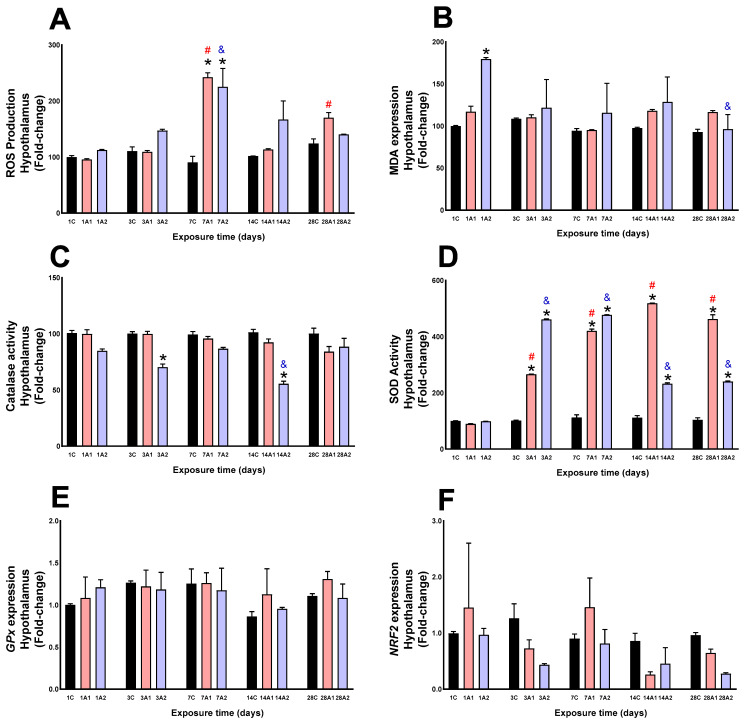
ROS production (**A**), MDA levels (**B**), Catalase activity (**C**), SOD activity (**D**), GPx expression (**E**) and NRF2 expression (**F**) in the rat hypothalamus following exposure to environmental hypoxia at two altitudes: A1 (3151 m), and A2 (4214 m), for 1, 3, 7, 14 and 28 days. Data are expressed as a percentage of control values and presented as mean ± SEM. Significant differences between A1 (red bars), A2 (blue bars) and control (black bars) groups at each time point are indicated by * *p* ≤ 0.05. Significant differences due to exposure duration effects within each altitude are indicated by ^#^ *p* ≤ 0.05 respect to day 1A1, and ^&^ *p* ≤ 0.05 respect to day 1A2. Statistical analysis was performed using two-way ANOVA followed by Tukey’s post hoc test.

In this study, ROS production in rat hypothalamus was evaluated following exposure to high (A1: 3151 m) and very high (A2: 4214 m) environmental hypoxia for 1, 3, 7, 14, and 28 days. Compared to sea level controls (C), a significant increase in ROS production was observed only on day seven (*7A1: 168% and *7A2: 150%, compared to control 7C). When analyzed by exposure duration, significant elevations in ROS were detected on days ^#^7A1 (153%) and ^#^28A1 (78%), compared to day one of exposure (1A1), and on day ^#^7A2 (100%), compared to day one of exposure (1A2) (Figure 1A). MDA levels showed a significant increase only on day *1A2 (79%), compared to control (1C). By exposure time, a significant decrease was observed on day ^&^28A2 (47%) compared to day one of exposure (1A2) (Figure 1B). Catalase activity significantly decreased at day *3A2 (30%) respect to control (3C), and at day *14A2 (46%) respect to control (14C). By exposure time, a significant decrease was observed on day ^&^14A2 (35%) compared to day one of exposure (1A2) (Figure 1C). SOD activity significantly increased by altitude at day *3A1 (163%) and *3A2 (356%) respect to control (3C), day *7A1 (275%) and *7A2 (325%) respect to control (7C), day *14A1 (366%) and *14A2 (109%) respect to control (14C), and day *28A1 (348%) and *28A2 (133%) respect to control (28C); when analyzed by exposure duration, significant increases in SOD levels were detected at ^#^3A1 (199%), ^#^7A1 (372%), ^#^14A1 (482%) and ^#^28A1 (419%) compared to control (1A1), and at ^&^3A2 (365%), ^&^7A2 (381%), ^&^14A2 (134%) and ^&^28A2 (142%) compared to control (1A2) (Figure 1D). The molecular expression of GPx (Figure 1E) and NRF2 (Figure 1F) showed no significant changes as a result of altitude and exposure time to environmental hypoxia.

**Figure 2:**
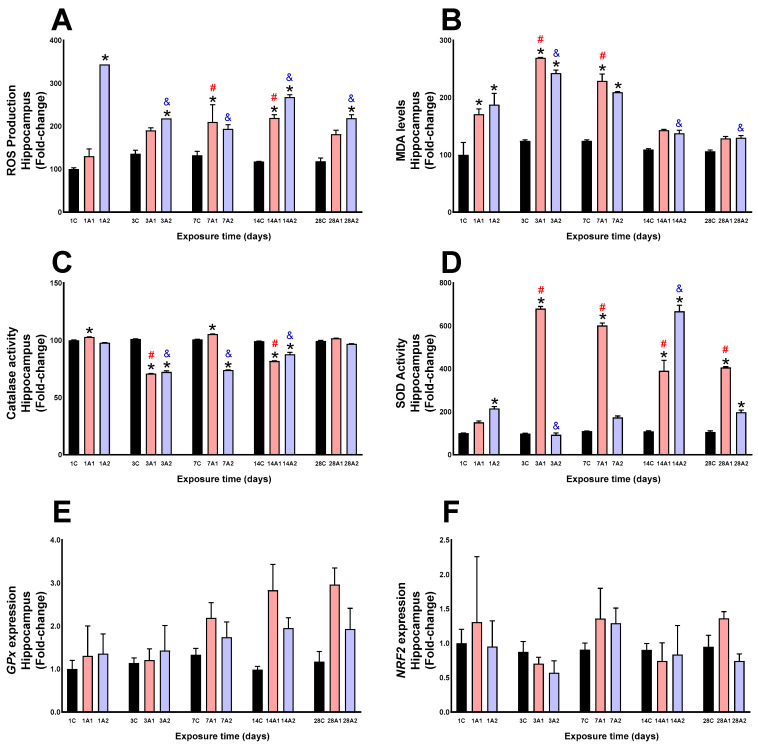
ROS production (**A**), MDA levels (**B**), Catalase activity (**C**), SOD activity (**D**), GPx expression (**E**) and NRF2 expression (**F**) in rat hippocampus following exposure to environmental hypoxia at two altitudes: A1 (3151 m), and A2 (4214 m), for 1, 3, 7, 14 and 28 days. Data are expressed as a percentage of control values and presented as mean ± SEM. Significant differences between A1 (red bars), A2 (blue bars) and control (black bars) groups at each time point are indicated by * *p* ≤ 0.05. Significant differences due to exposure duration effects within each altitude are indicated by ^#^ *p* ≤ 0.05 respect to day 1A1, and ^&^ *p* ≤ 0.05 respect to day 1A2. Statistical analysis was performed using two-way ANOVA followed by Tukey’s post hoc test.

In the hippocampus, ROS production significantly increased under the following conditions: *1A2 (244%) respect to control (1C), *3A2 (60%) respect to control (3C), *7A1 (58%) respect to control (7C), *14A1 (87%) and *14A2 (128%) respect to control (14C), and *28A2 (85%) respect to control (28C). By exposure duration, ROS levels were significantly elevated at days ^#^7A1 (60%) and ^#^14A1 (68%) compared to control (1A1), while ROS levels were significantly decreased at days ^&^3A2 (37%), ^&^7A2 (44%), ^&^14A2 (23%), and ^&^28A2 (37%) compared to control (1A2) (Figure 2A). Altitude significantly increased MDA levels at days *1A1 (70%) and *1A2 (87%) respect to control (1C), *3A1 (116%) and *3A2 (95%) respect to control (3C), *7A1 (84%) and *7A2 (68%) respect to control (7C). By exposure time, increases were found at day ^#^3A1 (58%) and ^#^7A1 (34%) respect to control (1A1), and ^&^3A2 (29%) respect 1A2, and decreased at days ^&^14A2 (27%) and ^&^28A2 (31%) compared to control (1A2) (Figure 2B). Altitude caused significant decreases in catalase activity at days *3A1 (31%) and *3A2 (29%) respect to control (3C), day *7A2 (26%) compared to 7C, 14A1 (19%) and *14A2 (13%) compared to 14 C, and slight increases at days *1A1 (2%) and *7A1 (5%). Time-course analysis showed decreases at days ^#^3A1 (32%) and ^#^14A1 (21%) respect to 1A1, and ^&^3A2 (27%), ^&^7A2 (25%) and ^&^14A2 (12%) compared to 1A2 (Figure 2C). Altitude significantly increased SOD activity at days *1A2 (115%) respect 1C, *3A1 (593%) respect to 3C, *7A1 (451%) respect to 7C, *14A1 (257%) and *14A2 (511%) respect to 14C, and *28A1, (286%) and *28A2 (88%) respect to 28C. Time-dependent increases were observed at days ^#^3A1 (350%), ^#^7A1 (298%), ^#^14A1 (158%), and ^#^28A1 (169%) compared to 1A1, and ^&^14A2 (210%) compared to 1A2 (Figure 2D). The molecular expression of GPx (Figure 2E) and NRF2 (Figure 2F) showed no significant changes as a result of altitude and exposure time to environmental hypoxia.

**Figure 3:**
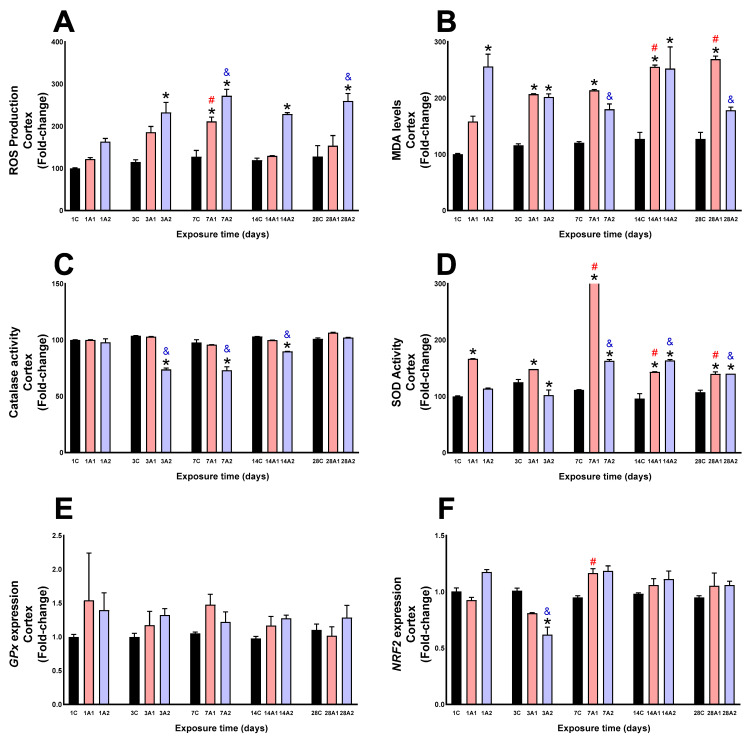
ROS production (**A**), MDA levels (**B**), Catalase activity (**C**), SOD activity (**D**), GPx expression (**E**) and NRF2 expression (**F**) in rat cortex following exposure to environmental hypoxia at two altitudes: A1 (3151 m), and A2 (4214 m), for 1, 3, 7, 14 and 28 days. Data are expressed as a percentage of control values and presented as mean ± SEM. Significant differences between A1 (red bars), A2 (blue bars) and control (black bars) groups at each time point are indicated by * *p* ≤ 0.05. Significant differences due to exposure duration effects (within each altitude) are indicated by ^#^ *p* ≤ 0.05 respect to day 1A1, and ^&^ *p* ≤ 0.05 respect to day 1A2. Statistical analysis was performed using two-way ANOVA followed by Tukey’s post hoc test.

In the cortex, significant increases in ROS were observed on days 3A2 (101%) respect to 3C, 7A1 (66%) and 7A2 (114%) respect to 7C, 14A2 (91%) respect to 14C, and 28A2 (102%) respect to 28C. ROS levels were increased by exposure duration at #7A1 (72%) compared to 1A1, and ^&^7A2 (67%) and ^&^28A2 (59%) compared to 1A2 (Figure 3A). MDA levels significantly increased due to the altitude at days *1A2 (156%) respect to 1C, *3A1 (79%) and *3A2 (74%) respect to 3C, *7A1 (77%) respect to 7C, *14A1 (100%) and *14A2 (98%) respect to 14C, and day *28A1 (111%) respect to 28C. By time of exposure, MDA levels increased at days ^#^14A1 (61%) and ^#^28A1 (70%) compared to 1A1, and decreased at days ^&^7A2 (30%), ^&^28A2 (31%) compared to 1A2 (Figure 3B). Catalase activity significantly decreased at days *3A2, (30%) respect to 3C, *7A2 (25%) respect to 7C, and *14A2 (13%) respect to 14C. Time-related decreases were observed at days ^&^3A2 (25%), ^&^7A2 (26%), and ^&^14A2 (8%) compared to 1A2 (Figure 3C). SOD activity significantly increased at days *1A1 (66%) respect to 1C, *3A1 (18%) respect to 3C, *7A1 (401%) and *7A2 (46%) respect to 7C, *14A1 (48%) and *14A2 (69%) respect to 14C, and *28A1 (30%) and *28A2 (30%) respect to 28C. A decrease was noted at day *3A2 (19%) respect to 3C. Time-dependent increases occurred at days ^#^7A1 (235%) respect to 1A1, and decreased at days ^#^14A1 (14%) and ^#^28A1 (16%) respect to 1A1, and ^&^7A2 (44%), ^&^14A2 (44%), and ^&^28A2 (24%) compared to 1A2 (Figure 3D). No changes were observed in GPx molecular expression as a result of altitude and exposure time to environmental hypoxia (Figure 3E). NRF2 expression decreased significantly at day *3A2 (38%) compared to 3C, and due to the effect of exposure time, a significant increase was observed at day ^#^7A1 (25%) respect to 1A1, and a significant decrease was observed at day ^&^3A2 (47%) respect to 1A2 of exposure to environmental hypoxia (Figure 3F).

**Figure 4:**
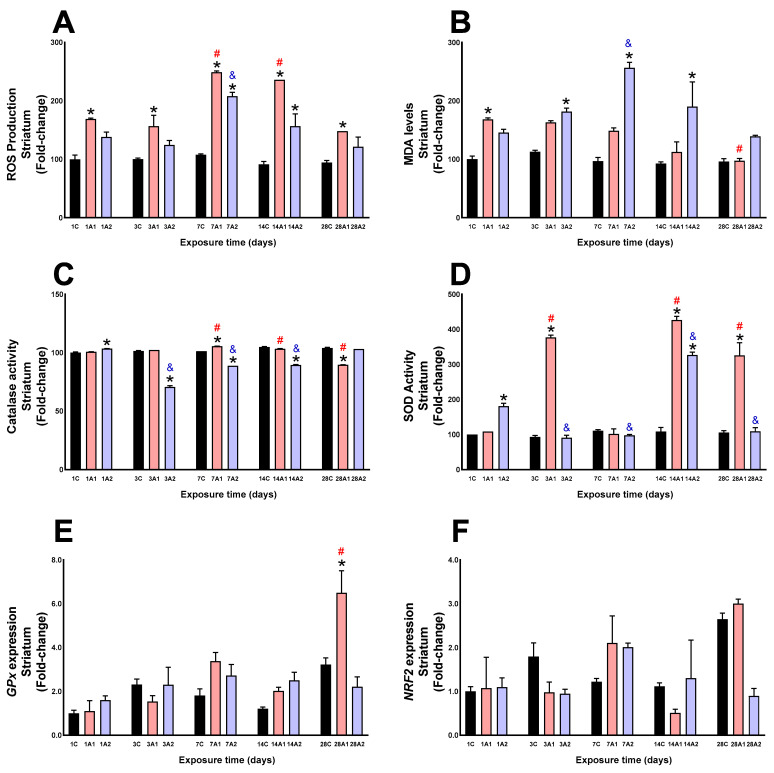
ROS production (**A**), MDA levels (**B**), Catalase activity (**C**), SOD activity (**D**), GPx expression (**E**) and NRF2 expression (**F**) in rat striatum following exposure to environmental hypoxia at two altitudes: A1 (3151 m), and A2 (4214 m), for 1, 3, 7, 14 and 28 days. Data are expressed as a percentage of control values and presented as mean ± SEM. Significant differences between A1 (red bars), A2 (blue bars) and control (black bars) groups at each time point are indicated by * *p* ≤ 0.05. Significant differences due to exposure duration effects (within each altitude) are indicated by ^#^ *p* ≤ 0.05 respect to day 1A1, and ^&^ *p* ≤ 0.05 respect to day 1A2. Statistical analysis was performed using two-way ANOVA followed by Tukey’s post hoc test.

In the striatum, ROS production significantly increased at days *1A1 (68%) respect to 1C, *3A1 (56%) respect to 3C, *7A1 (132%) and *7A2 (94%) respect to 7C, *14A1 (159%) and *14A2 (71%) respect to 14C, and *28A1 (57%) respect to 28C. Exposure duration analysis revealed significant elevated ROS levels at days ^#^7A1 (47%) and ^#^14A1 (39%) compared to 1A1, and ^&^7A2 (51%) compared to 1A2 (Figure 4A). In the striatum, significant MDA increases were observed at days *1A1 (68%) respect to 1C, *3A2 (61%) respect to 3C, *7A2 (166%) respect to 7C, and *14A2 (106%) respect to 14C. Time-course analysis showed increased levels at day ^&^7A2 (75%) compared to 1A2, and decreased levels at day ^#^28A1 (42%) compared to 1A1 (Figure 4B). In the striatum, catalase activity decreased significantly at day *3A2 (31%) compared to 3C, *7A2 (13%) compared to 7C, *14A2 (15%) compared to 14C, and *28A1 (14%) compared to 28C, and increased at day *1A2 (3%) respect to 1C, and *7A1 (3%) respect 7C. Time-related decreases were observed at days ^&^3A2 (32%), ^&^7A2 (14%) and ^&^14A2 (14%) respect to 1A2; increases were noted at days ^#^7A1 (5%) and ^#^14A1 (3%) compared to 1A1, but they decreased at day ^#^28A1 (11%) compared to 1A1 (Figure 4C). In the striatum, altitude significantly increased SOD activity at days *1A2 (81%) compared to 1C, *3A1 (304%) compared to 3C, *14A1 (294%) and *14A2 (202%) respect to 14C, and *28A1, (200%). Time-related increases were seen at days ^#^3A1 (248%), ^#^14A1 (294%) and ^#^28A1 (201%) compared to 1A1, while decreases were observed at days ^&^3A2 (50%), ^&^7A2 (47%) and ^&^28A2 (40%) compared to 1A2 and increased at day ^&^14A2 (80%) compared to 1A2 (Figure 4D). In the striatum, a significant increase in GPx expression was observed at days *28A1 (101%) compared to 28C, and ^#^28A1 (490%) compared to 1A1 (Figure 4E). In the striatum, neither altitude nor exposure time produced significant changes in NRF2 expression (Figure 4F).

### 2.2. Survival/Death Cell Pathways

**Figure 5:**
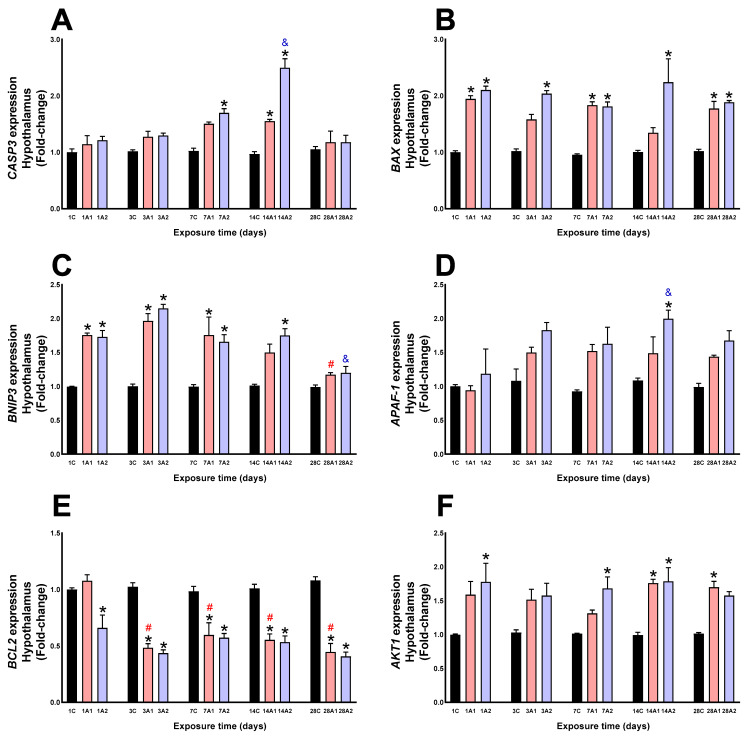
CASP3 (**A**), BAX (**B**), BNIP3 (**C**), APAF1 (**D**), BCL2 (**E**) and AKT1 (**F**) expression in rat hypothalamus following exposure to environmental hypoxia at two altitudes: A1 (3151 m), and A2 (4214 m), for 1, 3, 7, 14 and 28 days. Data are expressed as a percentage of control values and presented as mean ± SEM. Significant differences between A1 (red bars), A2 (blue bars) and control (black bars) groups at each time point are indicated by * *p* ≤ 0.05. Significant differences due to exposure duration effects within each altitude are indicated by ^#^ *p* ≤ 0.05 respect to day 1A1, and ^&^ *p* ≤ 0.05 respect to day 1A2. Statistical analysis was performed using two-way ANOVA followed by Tukey’s post hoc test.

In the hypothalamus, CASP3 expression significantly increased due to the effect of altitude (A1 and A2), on days *7A2 (69%) respect to 7C, *14A1 (55%) and *14A2 (149%) compared to 14C. With respect to exposure duration to environmental hypoxia, a significant increase was also noted at day ^&^14A2 (106%) compared to 1A2 (Figure 5A). BAX ex pression significantly increased under altitude conditions at days *1A1 (94%) and *1A2 (110%) respect to 1C, *3A2 (103%) respect to 3C, *7A1 (83%) and *7A2 (81%) respect to 7C, *14A2 (124%) respect to 14C, and *28A1 (77%) and *28A2 (88%) respect to 28C (Figure 5B). Altitude significantly increased BNIP3 expression at days *1A1 (75%) and *1A2 (72%) respect to 1C, *3A1 (96%) and *3A2 (115%) respect to 3C, *7A1 (75%) and *7A2 (65%) respect to 7C, and *14A2 (75%) respect to 14C. Exposure time caused a significant decrease at days ^#^28A1 (33%) compared to 1A1, and ^&^28A2 (30%) compared to 1A2 (Figure 5C). APAF-1 expression significantly increased at days 14A2 (*84%, ^&^69%) respect to 14C and 1A2, respectively (Figure 5D). BCL2 expression significantly decreased due to altitude at days *1A2 (34%) respect to 1C, *3A1 (53%) and *3A2 (58%) respect to 3C, *7A1 (40%) and *7A2 (42%) respect to 7C, *14A1 (45%) and *14A2 (47%) respect to 14C, and *28A1 (60%) and *28A2 (63%) respect to 28C; exposure time caused a significant decrease at days ^#^3A1 (55%), ^#^7A1 (45%), ^#^14A1 (49%), and ^#^28A1 (59%) respect to 1A1 (Figure 5E). By effect of altitude, AKT1 expression was significantly increased at days 1A2 (78%) compared to 1C, 7A2 (68%) compared to 7C, 14A1 (76%) and 14A2 (78%) compared to 14C, and 28A1 (70%) compared to 28C (Figure 5F).

**Figure 6:**
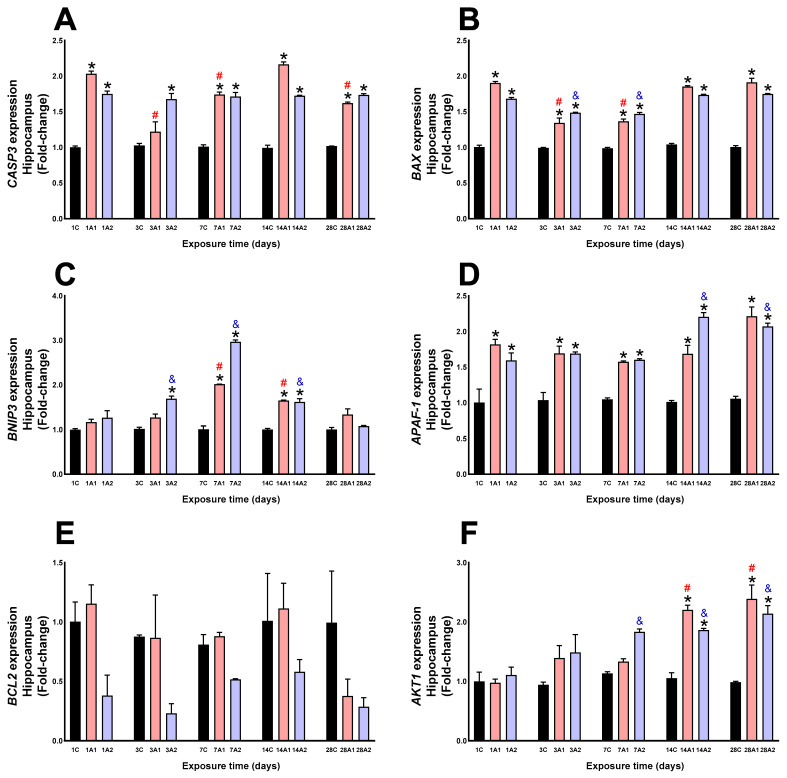
CASP3 (**A**), BAX (**B**), BNIP3 (**C**), APAF1 (**D**), BCL2 (**E**) and AKT1 (**F**) expression in rat hippocampus following exposure to environmental hypoxia at two altitudes: A1 (3151 m), and A2 (4214 m), for 1, 3, 7, 14 and 28 days. Data are expressed as a percentage of control values and presented as mean ± SEM. Significant differences between A1 (red bars), A2 (blue bars) and control (black bars) groups at each time point are indicated by * *p* ≤ 0.05. Significant differences due to exposure duration effects within each altitude are indicated by ^#^ *p* ≤ 0.05 respect to day 1A1, and ^&^ *p* ≤ 0.05 respect to day 1A2. Statistical analysis was performed using two-way ANOVA followed by Tukey’s post hoc test.

In the hippocampus, altitude significantly increased CASP3 expression at days *1A1 (103%) and *1A2 (75%) respect to 1C, *3A2 (67%) respect to 3C, *7A1 (74%) and *7A2 (71%) respect to 7C, *14A1 (116%) and *14A2 (72%) respect to 14C, and *28A1 (62%) and *28A2 (73%) respect to 28C. However, hypoxia duration led to a significant decrease at days ^#^3A1 (40%), ^#^7A1 (15%) and ^#^28A1 (20%) compared to 1A1 (Figure 6A). BAX expression increased with altitude at days *1A1 (90%) and *1A2 (68%) respect to 1C, *3A1 (34%) and *3A2 (48%) respect to 3C, 7A1 (36%) and *7A2 (47%) respect to 7C, *14A1 (85%) and 14A2 (73%) respect to 14C, and *28A1 (91%) and *28A2 (75%) respect to 28C. Exposure time led to a significant decrease at days ^#^3A1 (30%) and ^#^7A1 (29%) compared to 1A1, and ^&^3A2 (12%) and ^&^7A2 (12%) compared to 1A2 (Figure 6B). BNIP3 increased due to altitude at days *3A2 (68%) respect to 3C, *7A1 (101%) and *7A2 (196%) respect to 7C, *14A1 (65%) and *14A2 (61%) respect to 14C, and exposure time led to significant upregulation at days ^#^7A1 (73%) and ^#^14A1 (42%) compared to 1A1, and ^&^3A2 (33%), ^&^7A2 (135%) and ^&^14A2 (27%) compared to 1A2 (Figure 6C). APAF-1 expression increased significantly at days *1A1 (81%) and *1A2 (59%) respect to 1C, *3A1 (69%) and *3A2 (69%) respect to 3C, *7A1 (57%) and *7A2 (60%) respect to 7C, *14A1 (68%) and *14A2 (120%) respect to 14C, *28A1 (121%) and *28A2 (107%) respect to 28C. Exposure duration led to increased expression at days ^&^14A2 (38%) and ^&^28A2 (30%) compared to 1A2 (Figure 6D). Neither altitude nor exposure time produced significant changes in BCL2 expression (Figure 6E). Altitude increased AKT1 expression at days *14A1 (120%) and *14A2 (86%) compared to 14C, and *28A1 (139%) and *28A2 (114%) compared to 28C, and the effect time of exposure to environmental hypoxia was significantly increased at days ^#^14A1 (127%) and ^#^28A1 (146%) compared to 1A1, and ^&^7A2 (66%), ^&^14A2 (69%) and ^&^28A2 (94%) compared to 1A2 (Figure 6F).

**Figure 7:**
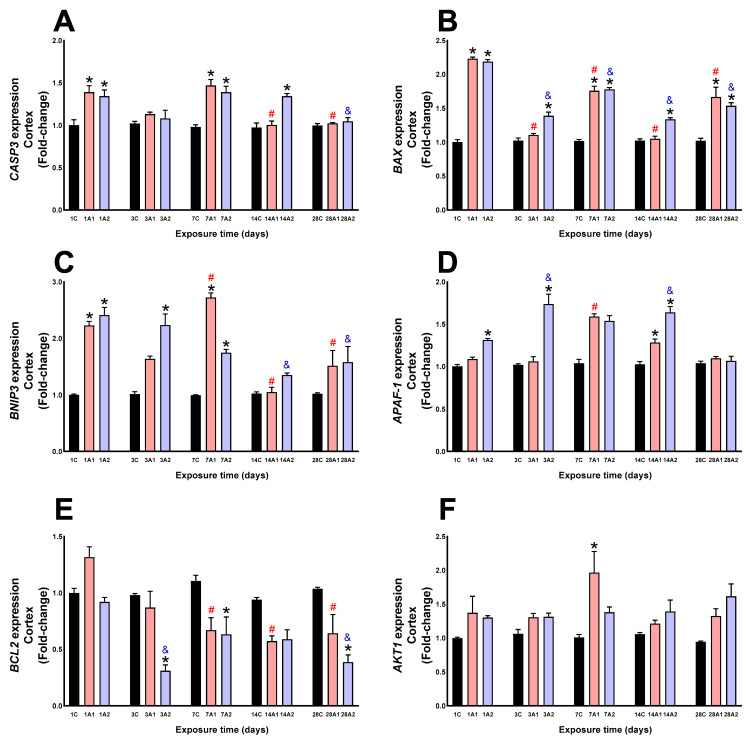
CASP3 (**A**), BAX (**B**), BNIP3 (**C**), APAF1 (**D**), BCL2 (**E**) and AKT1 (**F**) expression in rat cortex following exposure to environmental hypoxia at two altitudes: A1 (3200 m), and A2 (4214 m), for 1, 3, 7, 14 and 28 days. Data are expressed as a percentage of control values and presented as mean ± SEM. Significant differences between A1 (red bars), A2 (blue bars) and control (black bars) groups at each time point are indicated by * *p* ≤ 0.05. Significant differences due to exposure duration effects within each altitude are indicated by ^#^ *p* ≤ 0.05 respect to day 1A1, and ^&^ *p* ≤ 0.05 respect to day 1A2. Statistical analysis was performed using two-way ANOVA followed by Tukey’s post hoc test.

In the cortex, altitude caused a significant upregulation of CASP3 at days *1A1 (39%) and *1A2 (34%) respect to 1C, *7A1 (47%) and *7A2 (39%) respect to 7C, and *14A2 (34%) respect to 14C. However, prolonged hypoxia exposure led to a significant reduction in CASP3 expression at day ^#^14A1 (28%) and ^#^28A1 (27%) respect to 1A1, and ^&^28A2 (23%) respect to 1A2 (Figure 7A). Significant upregulation in BAX expression was observed at days *1A1 (123%) and *1A2 (119%) respect to 1C, *3A2 (39%) respect to 3C, *7A1 (76%) and *7A2 (78%) respect to 7C, *14A2 (33%) respect to 14C, and *28A1 (66%) and *28A2 (53%) respect to 28C. Exposure time significantly decreased BAX expression at days ^#^3A1 (51%), ^#^7A1 (21%), ^#^14A1 (53%) and ^#^28A1 (26%) respect to 1A1, and ^&^3A2 (37%), ^&^7A2 (19%), ^&^14A2 (40%) and ^&^28A2 (30%) respect to 1A2 (Figure 7B). BNIP3 expression was significantly upregulated by altitude at days *1A1 (123%) and *1A2 (141%) respect to 1C, *3A2 (123%) respect to 3C, and *7A1 (172%) and *7A2 (74%) respect to 7C. However, exposure time increased expression at day ^#^7A1 (22%) but caused reductions at days ^#^14A1 (53%) and ^#^28A1 (32%) compared to 1A1, and caused reductions at days ^&^14A2 (44%) and ^&^28A2 (35%) respect to 1A2 (Figure 7C). Altitude exposure increased APAF-1 expression at days *1A2 (31%) respect to 1C, *3A2 (71%) respect to 3C, *14A1 (25%) and *14A2 (61%) respect to 14C. Exposure duration significantly increased expression at day ^#^7A1 (46%) respect to 1A1, and ^&^3A2 (33%) and ^&^14A2 (25%) respect to 1A2 (Figure 7D). BCL2 expression significantly decreased at days *3A2 (68%) respect to 3C, *7A2 (43%) respect to 7C, and *28A2 (63%) respect to 28C. Exposure duration significantly decreased expression at days ^#^7A1 (49%), ^#^14A1 (56%) and ^#^28A1 (51%) respect to 1A1, and ^&^3A2 (66%) and ^&^28A2 (59%) respect to 1A2 (Figure 7E). Altitude (A1 and A2) significantly increased AKT1 expression at day 7A1 (94%) compared to control 7C (Figure 7F).

**Figure 8:**
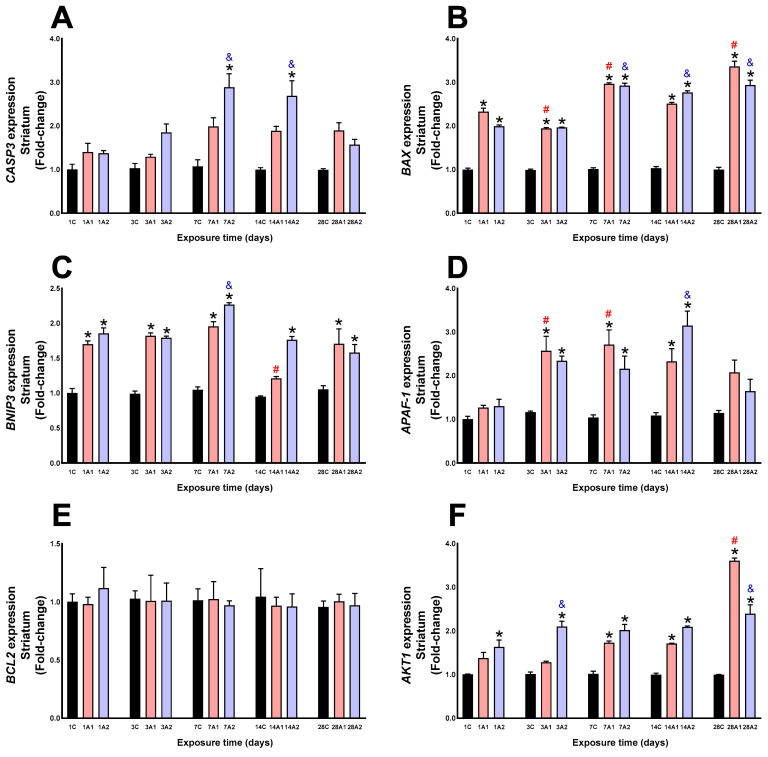
CASP3 (**A**), BAX (**B**), BNIP3 (**C**), APAF1 (**D**), BCL2 (**E**) and AKT1 (**F**) expression in rat striatum following exposure to environmental hypoxia at two altitudes: A1 (3151 m), and A2 (4214 m), for 1, 3, 7, 14 and 28 days. Data are expressed as a percentage of control values and presented as mean ± SEM. Significant differences between A1 (red bars), A2 (blue bars) and control (black bars) groups at each time point are indicated by * *p* ≤ 0.05. Significant differences due to exposure duration effects within each altitude are indicated by ^#^ *p* ≤ 0.05 respect to day 1A1, and ^&^ *p* ≤ 0.05 respect to day 1A2. Statistical analysis was performed using two-way ANOVA followed by Tukey’s post hoc test.

In the striatum, CASP3 expression increased significantly due to altitude at days *7A2 (169%) respect to 7C, and *14A2 (170%) respect to 14C. Exposure time also significantly increased CASP3 expression at day ^&^7A2 (110%) and ^&^14A2 (95%) respect to 1A2 (Figure 8A). BAX expression was significantly upregulated by altitude at days *1A1 (132%) and *1A2 (99%) respect to 1C, *3A1 (96%) and *3A2 (98%) respect to 3C, *7A1 (193%) and *7A2 (189%) respect to 7C, *14A1 (143%) and *14A2 (168%) respect to 14C, and *28A1 (236%) and *28A2 (193%) respect to 28C. Exposure time resulted in a significant increase at days ^#^7A1 (28%) and ^#^28A1 (45%) respect to 1A1, and ^&^7A2 (46%), ^&^14A2 (39%) and ^&^28A2 (47%) respect to 1A2, and a significant decrease at day ^#^3A1 (17%) respect to 1A1 (Figure 8B). Altitude increased BNIP3 at days *1A1 (70%) and *1A2 (85%) respect to 1C, *3A1 (84%) and *3A2 (81%) respect to 3C, *7A1 (88%) and *7A2 (117%) respect to 7C, *14A2 (87%) respect to 14C, *28A1 (62%) and *28A2 (50%) respect to 28C. Exposure time led to increased expression at day ^&^7A2 (22%) respect to 1A2, and decreased at day ^#^14A1 (29%) respect to 1A1 (Figure 8C). In the striatum, APAF-1 expression increased at days *3A1 (122%) and *3A2 (102%) respect to 3C, *7A1 (163%) and *7A2 (109%) respect to 7C, and *14A1 (115%) and *14A2 (191%) respect to 14C. Exposure time led to increased expression at days ^#^3A1 (103%) and ^#^7A1 (115%) respect to 1A1, and ^&^14A2 (142%) respect to 1A2 (Figure 8D). Neither altitude nor exposure time produced significant changes in BCL2 expression (Figure 8E). Altitude increased AKT1 expression at days *1A2 (63%) respect to 1C, *3A2 (110%) respect to 3C, *7A1 (70%) and 7A2 (99%) respect to 7C, *14A1 (73%) and *14A2 (111%) respect to 14C, *28A1 (260%) and *28A2 (139%) compared to 28C; due to the effect of time of exposure to environmental hypoxia, there was a significant increase at days ^#^28A1 (162%) respect to 1A1, and ^&^3A2 (29%) and ^&^28A2 (47%) respect to 1A2 (Figure 8F).

### 2.3. Neuroinflammation

**Figure 9:**
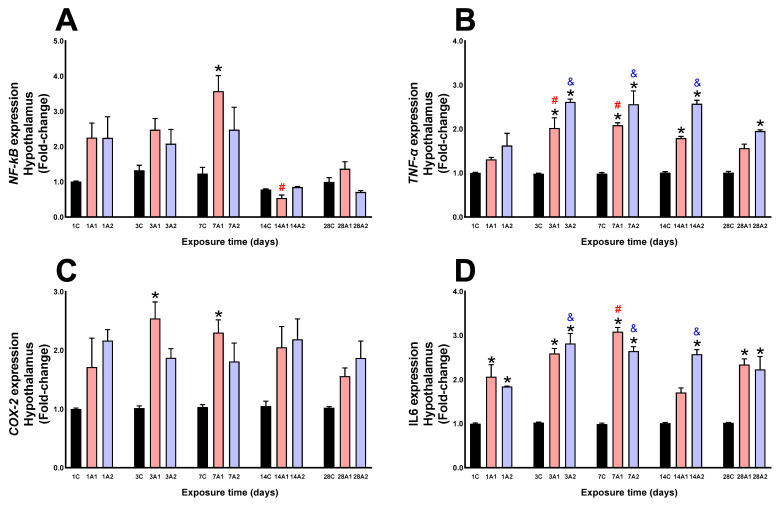
NF-kB (**A**), TNF-α (**B**), COX-2 (**C**) and IL6 (**D**) expression in rat hypothalamus following exposure to environmental hypoxia at two altitudes: A1 (3151 m), and A2 (4214 m), for 1, 3, 7, 14 and 28 days. Data are expressed as a percentage of control values and presented as mean ± SEM. Significant differences between A1 (red bars), A2 (blue bars) and control (black bars) groups at each time point are indicated by * *p* ≤ 0.05. While significant differences due to exposure duration effects within each altitude are indicated by ^#^ *p* ≤ 0.05 respect to day 1A1, and ^&^ *p* ≤ 0.05 respect to day 1A2. Statistical analysis was performed using two-way ANOVA followed by Tukey’s post hoc test.

In the hypothalamus, by effect of altitude, NF-kB expression increased significantly at day *7A1 (190%) compared to 7C, and by effect of time of exposure to environmental hypoxia, it decreased significantly at day ^#^14A1 (76%) respect to 1A1 (Figure 9A). TNF-α expression was significantly increased by altitude effect at days *3A1 (106%) and *3A2 (166%) respect to 3C, *7A1 (112%) and *7A2 (161%) respect to 7C, *14A1 (79%) and *14A2 (157%) respect to 14C, and *28A2 (95%) respect to 28C; by effect of time of exposure to environmental hypoxia, it increased significantly at days ^#^3A1 (54%) and ^#^7A1 (59%) respect to 1A1, and ^&^3A2 (61%), ^&^7A2 (58%) and ^&^14A2 (59%) respect to 1A2 (Figure 9B). COX-2 expression was significantly increased by altitude effect at days *3A1 (154%) respect to 3C, and *7A1 (123%) respect to 7C (Figure 9C). IL6 expression was significantly increased by altitude effect at days *1A1 (106%) and *1A2 (84%) respect to 1C, *3A1 (159%) and *3A2 (182%) respect to 3C, *7A1 (208%) and *7A2 (164%) respect to 7C, *14A2 (157%) respect to 14C, and *28A1 (134%) and *28A2 (123%) respect to 28C; by effect of time of exposure to environmental hypoxia, it increased significantly at days ^#^7A1 (50%) respect to 1A1, and ^&^3A2 (53%), ^&^7A2 (43%) and ^&^14A2 (40%) respect to 1A2 (Figure 9D).

**Figure 10:**
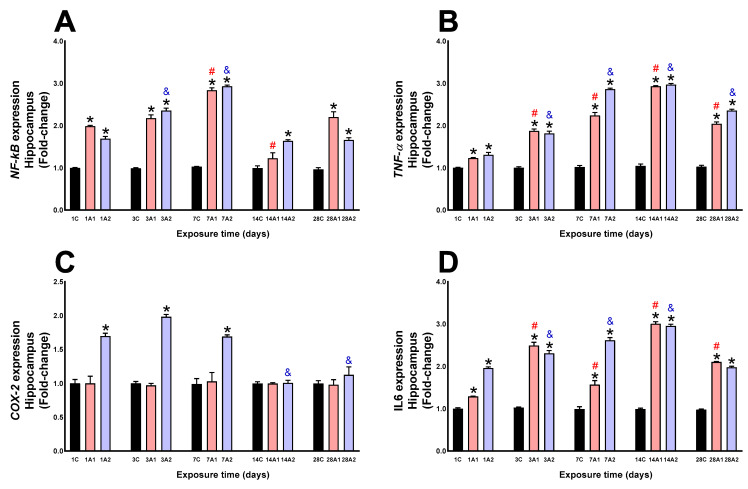
NF-kB (**A**), TNF-α (**B**), COX-2 (**C**) and IL6 (**D**) expression in rat hippocampus following exposure to environmental hypoxia at two altitudes: A1 (3151 m), and A2 (4214 m), for 1, 3, 7, 14 and 28 days. Data are expressed as a percentage of control values and presented as mean ± SEM. Significant differences between A1 (red bars), A2 (blue bars) and control (black bars) groups at each time point are indicated by * *p* ≤ 0.05. Significant differences due to exposure duration effects within each altitude are indicated by ^#^ *p* ≤ 0.05 respect to day 1A1, and ^&^ *p* ≤ 0.05 respect to day 1A2. Statistical analysis was performed using two-way ANOVA followed by Tukey’s post hoc test.

In the hippocampus, NF-kB expression was significantly increased by altitude effect at days *1A1 (98%) and *1A2 (69%) respect to 1C, *3A1 (117%) and *3A2 (135%) respect to 3C, *7A1 (183%) and *7A2 (193%) respect to 7C, *14A2 (63%) respect to 14C, and *28A1 (129%) and *28A2 (73%) respect to 28C; the effect of time of exposure to environmental hypoxia, significantly increased at days ^#^7A1 (43%) and ^#^14A1 (38%) respect to 1A1, and ^&^3A2 (39%) and ^&^7A2 (73%) respect to 1A2 (Figure 10A). TNF-α expression was significantly increased by altitude effect at days *1A1 (23%) and *1A2 (30%) respect to 1C, *3A1 (87%) and *3A2 (81%) respect to 3C, *7A1 (124%) and *7A2 (186%) respect to 7C, *14A1 (79%) and *14A2 (157%) respect to 14C, *28A1 (56%) and *28A2 (95%) respect to 28C; by effect of time of exposure to environmental hypoxia, it significantly increased at days ^#^3A1 (52%), ^#^7A1 (82%), ^#^14A1 (138%) and ^#^28A1 (66%) respect to 1A1, and ^&^3A2 (39%), ^&^7A2 (120%), ^&^14A2 (128%) and ^&^28A2 (81%) compared to 1A2 (Figure 10B). COX-2 expression was significantly increased by the effect of altitude at days *1A2 (69%) respect to 1C, and *3A2 (98%) respect to 3C and *7A2 (69%) respect to 7C; by the effect of time of exposure to environmental hypoxia, it was significantly decreased at day ^&^14A2 (41%) and ^&^28A2 (34%) respect to 1A2 (Figure 10C). IL6 expression was significantly increased by altitude effect at days *1A1 (29%) and *1A2 (96%) respect to 1C, *3A1 (149%) and *3A2 (130%) respect to 3C, *7A1 (57%) and *7A2 (161%) respect to 7C, *14A1 (200%) and *14A2 (195%) respect to 14C, and *28A1 (110%) and *28A2 (97%) respect to 28C; by effect of time of exposure to environmental hypoxia, it increased significantly at day ^#^3A1 (93%), ^#^7A1 (22%), day ^#^14A1 (132%) and ^#^28A1 (63%) compared to 1A1, and ^&^3A2 (17%), ^&^7A2 (33%), and ^&^14A2 (51%) respect to 1A2 (Figure 10D).

**Figure 11:**
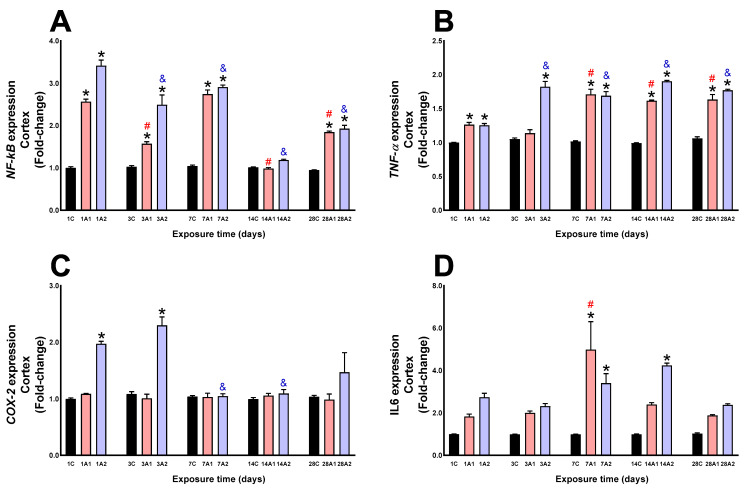
NF-kB (**A**), TNF-α (**B**), COX-2 (**C**) and IL6 (**D**) expression in rat cortex following exposure to environmental hypoxia at two altitudes: A1 (3151 m), and A2 (4214 m), for 1, 3, 7, 14 and 28 days. Data are expressed as a percentage of control values and presented as mean ± SEM. Significant differences between A1 (red bars), A2 (blue bars) and control (black bars) groups at each time point are indicated by * *p* ≤ 0.05. Significant differences due to exposure duration effects (within each altitude) are indicated by ^#^ *p* ≤ 0.05 respect to day 1A1, and ^&^ *p* ≤ 0.05 respect to day 1A2. Statistical analysis was performed using two-way ANOVA followed by Tukey’s post hoc test.

In the cortex, NF-kB expression was significantly increased by the effect of altitude at days *1A1 (156%) and *1A2 (241%) respect to 1C, *3A1 (57%) and *3A2 (149%) respect to 3C, *7A1 (174%) and *7A2 (190%) respect to 7C, and *28A1 (84%) and *28A2 (92%) respect to 28C; the effect of exposure time to environmental hypoxia decreased significantly on the days ^#^3A1 (39%), ^#^14A1 (62%) and ^#^28A1 (28%) compared to 1A1, and ^&^3A2 (27%), ^&^7A2 (15%), ^&^14A2 (65%) and ^&^28A2 (44%) compared to 1A2 (Figure 11A). TNF-α expression was significantly increased at days *1A1 (26%) and *1A2 (25%) respect to 1C, *3A2 (82%) respect to 3C, *7A1 (71%) and *7A2 (69%) respect to 7C, *14A1 (61%) and *14A2 (90%) respect to 14C, and *28A1 (63%) and *28A2 (77%) respect to 28C; by effect of time of exposure to environmental hypoxia, it significantly increased at days ^#^7A1 (36%), ^#^14A1 (28%) and ^#^28A1 (29%) compared to 1A1, and ^&^3A2 (46%), ^&^7A2 (35%), ^&^14A2 (52%) and ^&^28A2 (42%) compared to 1A2 (Figure 11B). In the cortex, the effect of altitude COX-2 expression was significantly increased at days *1A2 (97%) respect to 1C, and *3A2 (129%) respect to 3C; by effect of time of exposure to environmental hypoxia, it was significantly decreased at days ^&^7A2 (47%) and ^&^14A2 (45%) compared to 1A2 (Figure 11C). IL6 expression was significantly increased by altitude effect at day *7A1 (408%) and *7A2 (247%) compared to 7C, and *14A2 (332%) compared to 14C; by effect of time of exposure to environmental hypoxia, it increased significantly at day ^#^7A1 (174%) respect to 1A1 (Figure 11D).

**Figure 12:**
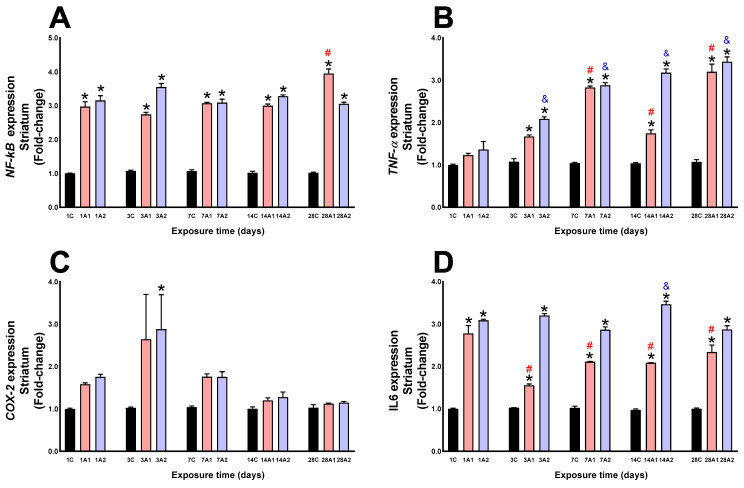
NF-kB (**A**), TNF-α (**B**), COX-2 (**C**) and IL6 (**D**) expression in rat striatum following exposure to environmental hypoxia at two altitudes: A1 (3151 m), and A2 (4214 m), for 1, 3, 7, 14 and 28 days. Data are expressed as a percentage of control values and presented as mean ± SEM. Significant differences between A1 (red bars), A2 (blue bars) and control (black bars) groups at each time point are indicated by * *p* ≤ 0.05. Significant differences due to exposure duration effects within each altitude are indicated by ^#^ *p* ≤ 0.05 respect to day 1A1, and ^&^ *p* ≤ 0.05 respect to day 1A2. Statistical analysis was performed using two-way ANOVA followed by Tukey’s post hoc test.

In the striatum, altitude (A1 and A2) produced a significant increase in NF-kB expression at days *1A1 (197%) and *1A2 (215%) respect to 1C, *3A1 (174%) and *3A2 (255%) respect to 3C, *7A1 (207%) and *7A2 (209%) respect to 7C, *14A1 (199%) and *14A2 (228%) respect to 14C, and *28A1 (295%) and *28A2 (205%) respect to 28C; due to the effect of exposure time to environmental hypoxia, a significant increase was found on day ^#^28A1 (33%) respect to 1A1 (Figure 12A). Altitude (A1 and A2) produced a significant increase in TNF-α expression at days *3A1 (67%) and *3A2 (108%) respect to 3C, *7A1 (182%) and *7A2 (188%) respect to 7C, *14A1 (74%) and *14A2 (217%) respect to 14C, and *28A1 (220%) and *28A2 (243%) respect to 28C; by effect of time of exposure to environmental hypoxia, a significant increase was found at days ^#^7A1 (129%), ^#^14A1 (41%) and ^#^28A1 (160%) compared to 1A1, and ^&^3A2 (53%), ^&^7A2 (112%), ^&^14A2 (133%) and ^&^28A2 (152%) compared to 1A2 (Figure 12B). Altitude (A2) produced a significant increase in COX-2 expression at day *3A2 (188%) compared to 3C (Figure 12C). IL6 expression was significantly increased by altitude effect (A1 and A2) at days *1A1 (177%) and *1A2 (208%) respect to 1C, *3A1 (55%) and *3A2 (220%) respect to 3C, *7A1 (110%) and *7A2 (186%) respect to 7C, *14A1 (108%) and *14A2 (246%) respect to 14C, and *28A1 (133%) and *28A2 (187%) respect to 28C; by effect of time of exposure to environmental hypoxia, it increased ^&^14A2 (12%) respect to 1A2, and decreased ^#^3A1 (44%), ^#^7A1 (24%), ^#^14A1 (25%) and ^#^28A1 (16%) respect to 1A1 (Figure 12D).

## 3. Discussion

The present study demonstrates that exposure to environmental hypoxia at high (3151 m) and very high (4214 m) altitudes induces significant oxidative stress, neuroinflammation, and alterations in cell death pathways across multiple brain regions in rats. Key findings include increased production of ROS and MDA, elevated SOD activity, and reduced catalase activity, collectively indicating an imbalance in the antioxidant defense system. These biochemical alterations were accompanied by overexpression of pro-apoptotic (CASP3, BAX, BNIP3, APAF1) and pro-inflammatory (NF-κB, TNF-α, COX-2 and IL6) genes, alongside a general reduction in anti-apoptotic markers such as BCL2. Importantly, these effects were both region- and time-dependent, with more pronounced changes observed at higher altitudes and after longer exposure durations.

Cellular function depends critically on oxygen availability, and fluctuations in oxygen concentration can disrupt homeostasis and compromise cell viability. Oxygen delivery varies across organs, and in the brain, the partial pressure of oxygen (pO_2_) is not uniform among regions [22,23]. High-altitude environments are characterized by reduced oxygen pressure, along with other stressors such as cold temperatures, low humidity, and increased radiation: factors that significantly impact brain physiology [24]. Chronic exposure to such conditions in structural brain changes, largely due to altered cerebral blood flow and increased BBB permeability [25,26], has been linked to cognitive impairments [27,28].

Hypoxia impairs mitochondrial oxidative phosphorylation by reducing electron transport chain efficiency, thereby enhancing ROS production and stimulating nitric oxide synthase activity. Depending on the intensity and duration of exposure, cells may adapt or undergo oxidative stress, cytotoxicity, or apoptosis. In response, hypoxia activates various genes involved in metabolic reprogramming and cellular remodeling [29].

ROS are natural byproducts of mitochondrial respiration, formed when electrons leak from the electron transport chain and react with molecular oxygen to form superoxide anions. Under normoxic conditions, ROS are generated at low levels and serve critical roles in cellular signaling [30,31,32,33]. However, paradoxically, several studies have reported elevated ROS production under hypoxic conditions [34,35]. Consistent with these findings, our study showed significantly increased ROS levels in the cortex, hippocampus, and striatum under both high (A1) and very high (A2) hypoxic conditions. ROS levels also increased progressively with prolonged exposure (3, 7, 14, and 28 days), supporting the hypothesis that sustained hypoxic stress enhances oxidative load. Excessive ROS disrupts cellular signaling and damages macromolecules, ultimately promoting cell death [36].

Previous studies have shown that ROS levels can rise rapidly within minutes of hypoxic exposure, peaking around 30 min and partially resolving after 48 h [37]. Individuals who are not acclimatized to high-altitude are particularly susceptible to oxidative stress, resulting in cellular dysfunction through free-radical-mediated damage to lipids, proteins, and DNA [38,39].

MDA, a lipid peroxidation byproduct, serves as a well-established marker of oxidative stress. In our study, MDA levels in the hypothalamus peaked on day one of very high hypoxia and declined by day 28. Conversely, MDA levels in the cortex remained elevated throughout the exposure period. The hippocampus exhibited significant increases on days 1, 3, and 7, whereas the striatum showed elevations on days 1, 3, 7, and 14. Previous studies have demonstrated similar patterns, with increased MDA observed between 6 and 48 h of hypoxic exposure [40,41]. Moreover, very high altitudes (>5000 m) have been associated with imbalances between MDA concentration and antioxidant enzyme activity, particularly SOD, highlighting the oxidative burden of such environments [42].

Antioxidant enzymes such as SOD and catalase are essential for maintaining redox balance. SOD catalyzes the conversion of superoxide radicals into hydrogen peroxide, which is further broken down into water and oxygen by catalase. In our study, SOD activity increased while catalase activity decreased in all brain regions, particularly under very high hypoxia. These findings suggest an imbalance in the antioxidant defense system. The literature reports on the effects of hypoxia on these enzymes remain mixed. Some studies found no significant changes in SOD or catalase levels in rat plasma after 22 days at 5000 m [43], while others reported increased SOD but reduced catalase in the heart [44]. Conversely, reduced SOD activity has been observed in the lungs after seven days at 4200 m [45], and both SOD and catalase levels decreased in plasma and lung tissues under simulated high-altitude conditions [46,47]. Notably, brain SOD levels have been shown to decrease after short-term exposure (5 h) to 9144 m [47] and after 24–48 h at 7620 m [40,48].

Environmental hypoxia induces a redox imbalance in the nervous system, as demonstrated in the present study, that is characterized by increased production of ROS at the mitochondrial level, which triggers a compensatory response from endogenous antioxidant systems, particularly SOD and catalase. SOD, together with catalase, converts O_2_^−^• into water and oxygen, preventing its accumulation and the generation of highly toxic hydroxyl radicals. However, under conditions of sustained or severe hypoxia, continuous ROS overproduction can saturate the capacity of these enzymes, reducing their activity and leading to a state of oxidative stress that compromises neuronal integrity and brain homeostasis. Studies in experimental models show that, although SOD and catalase activity may initially increase as an adaptive mechanism, prolonged exposure to low partial oxygen pressure ultimately reduces their effectiveness due to the oxidation of functional groups and alterations in gene expression regulated by factors such as NF-κB and Nrf2, which promote neurodegenerative processes and cognitive dysfunction [49].

We also assessed the expression of key apoptotic markers, including CASP3, BAX, BNIP3, APAF1, BCL2, and AKT1. With the exception of BCL2, all genes related to cell death showed significant upregulation in the hypothalamus, cortex, hippocampus, and striatum in response to altitude (A1 and A2) and exposure duration (1–28 days). These results align with previous findings of elevated CASP3 activity following 6 h of very high hypoxia [7,50], and increased CASP3-mediated apoptosis in PC12 cells under hypobaric hypoxia [51]. Hypobaric hypoxia (8000 m) has been shown to impair mitochondrial function and induce neuronal apoptosis via upregulation of CASP3, and BAX and downregulation of BCL2 [52,53]. These processes have been validated by histological and immunofluorescence studies [54,55] and confirmed by transcriptomic analyses in hypoxia-exposed mice [56].

Under hypoxic conditions, the activation of intrinsic and mitochondrial apoptotic pathways is reflected in the coordinated increase in multiple proapoptotic markers. Increased BAX expression promotes permeabilization of the outer mitochondrial membrane, which facilitates the release of cytochrome c into the cytosol. This event enables the formation of the apoptosome through the interaction of APAF1 with cytochrome c and procaspase-9, triggering the sequential activation of CASP3. Similarly, BNIP3 overexpression can be induced, exacerbating mitochondrial dysfunction. The overexpression of these cell death biomarkers suggests that environmental hypoxia not only activates adaptive signals but also drives a robust apoptotic program, probably as a mechanism to eliminate damaged cells or those unable to maintain energy homeostasis under low O_2_ conditions [57,58]. This molecular pattern is consistent with the data obtained in our study.

In parallel, we observed an increased expression of inflammation-related genes (NF-kB, TNF-α, IL-6, and COX-2), and a decreased expression of antioxidant genes (NRF2 and GPx) under both A1 and A2 conditions, especially with prolonged exposure. Similar patterns have been reported in the hippocampus of mice exposed to 7000 m for one to seven days, showing elevated NF-kB, COX2, IL6 and TNF-α and decreased GPx expression [59,60]. Hypoxia-induced inflammation has also been documented in the lungs (6500 m) and hearts (6000 m) of rats, marked by increased TNF-α and IL6, and reduced NRF2 expression [61,62,63]. Interestingly, one study found concurrent increases in NRF2, TNF-α, and NF-κB in the brains of mice exposed to 8000 m, reflecting the complex interplay between oxidative stress and inflammatory responses [64].

Environmental hypoxia causes significant activation of pro-inflammatory pathways mediated by NF-κB in brain tissue, triggering the increased transcription of genes such as TNF-α and IL-6, as well as pro-inflammatory enzymes such as COX-2. Hypoxia induces oxidative stress and mitochondrial dysfunction that activates NF-κB, which enhances the brain’s inflammatory response [65]. The increase in TNF-α and IL-6 contributes to BBB alteration, promoting immune cell infiltration and aggravating neuronal damage [66]. Furthermore, COX-2 overexpression in neurons and glial cells under hypoxic conditions is associated with increased production of pro-inflammatory prostaglandins and the progression of neurodegenerative processes [67]. These findings support the concept that environmental hypoxia acts as a potent inflammatory stimulus in the brain, mediated by the coordinated activation of NF-κB and other inflammatory cytokines.

In this study, we did not evaluate cognitive or motor behaviors resulting from environmental hypoxia, nor did we observe any external behaviors in the animals that could indicate motor-cognitive alterations. However, we believe that the exacerbation of biomarkers of oxidative stress, inflammation, and cell death could alter motor-cognitive regulation at the brain level. In various memory studies, significant deterioration in working memory, spatial memory, and long-term memory has been documented, which is attributed to the high sensitivity of the hippocampus to hypoxia. This condition also interferes with the acquisition of new information and reduces overall cognitive performance, affecting executive functions such as sustained attention, sensory processing, and decision-making [68,69]. These effects are intensified with prolonged or repeated exposure to altitude, compromising synaptic plasticity and hippocampal-prefrontal axis activity [70].

Regarding motor skills, altitude hypoxia can cause alterations in fine motor coordination, slowed reaction times, and impaired performance of complex motor tasks due to dysfunction of brain structures, such as the cerebellum and motor cortex [70,71]. These effects are mediated by mechanisms such as oxidative stress.

Collectively, our findings demonstrate that environmental hypoxia triggers a cascade of molecular events—including oxidative stress, inflammation, and apoptosis—across multiple brain regions. The severity of these changes depends on both altitude and exposure duration. These results suggest that high-altitude hypoxia may be a contributing factor to neurological impairments, including cognitive decline, behavioral abnormalities, and the development of neurodegenerative disorders.

## 4. Materials and Methods

### 4.1. Chemicals

This study used materials of the highest quality and purity. The main reagents used were DCFH-DA, HEPES, sucrose, and protease inhibitors (Sigma-Aldrich, Saint Louis, MO, USA) for ROS production assays. Sodium dodecyl sulfate and thiobarbituric acid (Sigma-Aldrich, Saint Louis, MO, USA) were used for the MDA assay. Catalase activity kit and SOD activity kit (Invitrogen, Thermo Fisher Scientific, Waltham, MA, USA) were used for antioxidant enzyme assays. RNA extraction Nucleospin RNA plus kit (Macherey-Nagel, Düren, Germany) was used for RNA extraction. cDNA UltraScript^®®^ Separate Oligos kit (PCRBIOSYSTEMS, London, UK) was used for reverse transcription. FastGene IC green Universal MIX Master Mix (Nippon Genetics, Düren, Germany) was used for quantitative PCR. The target genes and the specific primers (Sigma-Aldrich, Saint Louis, MO, USA) were the following:
**Target Gene****Forward Primer****Reverse Primer***GAPDH*5′-TCCCTGTTCTAGAGACAG-3′5′-CCACTTTGTCACAAGAGA-3′*BCL2*5′-GTACCTGAACCGGCATCTG-3′5′-GGGGCCATATAGTTCCACAA-3′*CASP3*5′-AATTCAAGGGACGGGTCATG-3′5’-GCTTGTGCGCGTACAGTTTC-3′*BAX*5′-CTGCAGAGGATGATTGCTGA-3′5′-GATCAGCTCGGGCACTTTAG-3′*BNIP3*5′-TTTTAAACACCCGAAGCGCA-3′5′-TGAGCAGAAGGCAGATCCAA-3′*APAF1*5′-TTCAGGTTTGTAGCTCGGCA-3′5′-ACCCAAGGATCCCAAACGTC-3′*AKT1*5′-CACCGCTTCTTTGCCAACAT-3′5′-CACACACTCCATGCTGTCATCT-3′*NRF2*5′-TGTAGATGACCATGAGTCGC-3′5′- TGTCCTGCTGTATGCTGCTT-3′*GPx*5′-TCAGTTCGGACATCAGGAGA-3′5′-GAAGGTAAAGAGCGGGTGAG-3′*COX2*5′-CAGGAGAGAAAGAAATGGCTGC-3′5′-TGGTCTCCCCAAAGATAGCATC-3′*TNF-α*5′-ATCCGAGATGTGGAACTGGC-3′5′-AAATGGCAAATCGGCTGACG-3′*NFκB*5′-ATATTCACCTGCACGCCCAC-3′5′-GGTTTGCAAAGCCAACCACC-3′

Data were normalized to the expression of the housekeeping gene GAPDH and calculated by the ΔΔCt (Delta Delta Ct) method.

### 4.2. Animals and Sample Collection

All experimental procedures were reviewed and approved by the Animal Welfare Ethics Committee of the Faculty of Veterinary Medicine of the National University of San Marcos (authorization CEBA 2021-21). The animals came from the National Institute of Health in Lima, Peru. They were acquired one week before the start of the study to allow for habituation. After this week of habituation, the animals were transferred to the two high-altitude locations, located less than three hours from Lima.

A total of 75 male albino rats (8 weeks old, weighing approximately 180 g) were used in this study. Animals were housed individually in polycarbonate cages with sawdust bedding under controlled environmental conditions: temperature of 22 ± 1 °C, relative humidity of 40%, and 12 h light/dark cycle. Standard rodent chow and water were provided ad libitum.

**Figure 13:**
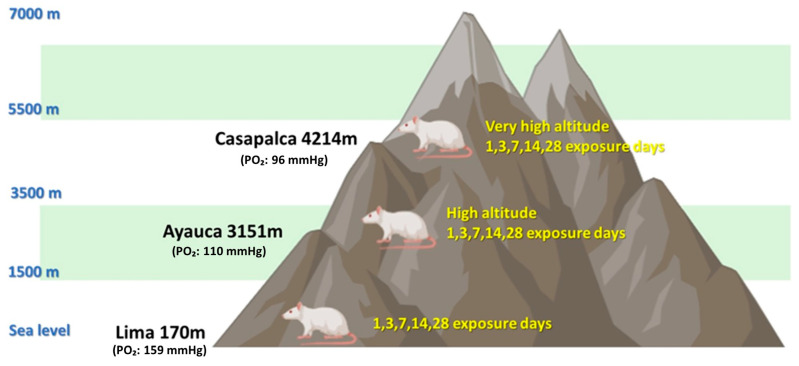
Time (1, 3, 7, 14, and 28 days) of exposure to environmental hypoxia and geographical locations where the rats were raised. Control group (C, sea level), exposed to high altitude (A1, 3151 m) and very high altitude (A2, 4214 m).

The animals were randomly assigned to 15 groups (n = 5 per group):

- Five control groups (**C**; 1, 3, 7, 14 and 28 days of exposure) maintained at sea level (170 m, PO_2_: 159 mmHg, Lima, Peru).
- Five groups exposed to high altitude **A1** (3151 m, PO_2_: 110 mmHg, Ayauca-Yauyos, Peru) for 1, 3, 7, 14, or 28 days.
- Five groups exposed to very high altitude **A2** (4214 m, PO_2_: 96 mmHg, Casapalca-Huarochirí, Peru) for the same time periods (Figure 13).

At each designated time point (1, 3, 7, 14, and 28 days) in the respective breeding locations, animals were euthanized by decapitation, and their brains were rapidly dissected on ice (at 4 °C) into four distinct regions: hypothalamus, cortex, hippocampus, and striatum [72]. All tissue samples were immediately frozen at −80 °C for subsequent biochemical and molecular analyses and transported to laboratories in Lima (three hours distance) under appropriate cold-chain conditions.

### 4.3. Oxidative Stress Assay (ROS Production)

To evaluate ROS production, 10 mg of tissue from each brain region was homogenized in a buffer containing 50 mM HEPES, 320 mM sucrose, and protease inhibitors. A 15 µL aliquot of the homogenate (equivalent to 1 mg tissue) was mixed with 80 µM DCFH-DA (dissolved in DMSO) in phosphate-buffered saline (PBS). The mixture was incubated in black 96-well plates at 37 °C for 30 min [73]. Fluorescence intensity was measured using an FLX800 fluorometer (BioTek, Winooski, VT, USA) at excitation/emission wavelengths of 485 nm/528 nm.

### 4.4. Malondialdehyde (MDA) Assay

Lipid peroxidation levels were assessed by measuring malondialdehyde (MDA). A total of 10 mg of brain tissue were homogenized with a lysis buffer containing sodium dodecyl sulfate (SDS), followed by centrifugation at 13,000× *g* for 15 min at 4 °C. A 250 μL aliquot of the supernatant was mixed with 250 μL of thiobarbituric acid (0.07%) in sodium sulfate solution [74]. The mixture was incubated at 95 °C for 60 min and absorbance was measured at 532 nm using a spectrophotometer (Agilent Technologies, Santa Clara, CA, USA).

### 4.5. Catalase Enzyme Activity Assay

Catalase activity was determined using a colorimetric assay kit (Invitrogen, Thermo Fisher Scientific, MA, USA) following the manufacturer’s protocol. Brain tissues (≥30 mg) were washed with PBS and homogenized in 0.5 mL of cold 1× assay buffer. The homogenate was centrifuged at 10,000× *g* for 15 min at 4 °C (Eppendorf 5702R, Leipzig, Germany), and the supernatant was used for colorimetric measurement at 560 nm (Agilent Technologies, CA, USA).

### 4.6. Superoxide Dismutase (SOD) Enzyme Activity Assay

SOD activity was assessed using a colorimetric assay kit (Invitrogen, Thermo Fisher Scientific, MA, USA) following the manufacturer’s instructions. Brain tissues (≥30 mg) were washed with cold PBS and homogenized in 0.5 mL of cold PBS. The homogenates were centrifuged at 1500× *g* for 10 min at 4 °C (Eppendorf 5702R, Leipzig, Germany) and the supernatants were analyzed by spectrophotometry (Agilent Technologies, CA, USA).

### 4.7. Molecular Analysis

Each brain sample (30 mg) underwent three sequential steps: total RNA extraction, cDNA synthesis, and quantitative PCR (qPCR).

Total RNA was extracted, using Nucleospin RNA plus extraction kit (Macherey-Nagel, Germany) following the manufacturer’s protocol. RNA purity was confirmed with 260/280 >1.95. Complementary DNA (cDNA) was synthesized, using the UltraScript^®®^ Separate Oligos kit (PCRBIOSYSTEMS, London, UK), and stored at −80 °C for subsequent analysis.

Quantitative real-time PCR (qPCR) was performed using the FastGene IC green Universal MIX Master Mix (Nippon Genetics, Germany), optimized for amplicons between 80 and 300 bp with a melting temperature of 60 °C. The cycling conditions were as follows: initial denaturation at 95 °C for 2 min (1 cycle); denaturation at 95 °C for 5 s (40 cycles); annealing/elongation at 60–65 °C for 20–30 s (40 cycles) [75].

### 4.8. Statistical Analysis

Data were analyzed using GraphPad Prism 8.0.1 (GraphPad software, Boston, MA, USA). Results were normalized to the control and are presented as mean ± standard error of the mean (SEM).

Two-way analysis of variance (ANOVA) was employed to evaluate the effects of altitude (C, A1 and A2) and exposure time to environmental hypoxia (1, 3, 7, 14 and 28 days), followed by Tukey’s post hoc test for multiple comparisons. Significant differences attributed to altitude (C vs. A1 or A2, at each time point) are indicated by * *p* < 0.05. Statistically significant differences due to exposure time at 3151 m are indicated by ^#^ *p* < 0.05, taking day 1A1 as a reference, and statistically significant differences due to the duration of exposure at 4214 m are indicated by ^&^ *p* < 0.05, taking day 1A2 as a reference.

## 5. Conclusions

Collectively, these results underscore the critical role of oxidative stress and neuroinflammation in the pathophysiology of high-altitude-induced brain injury. The findings suggest that hypoxia disrupts redox homeostasis and increases neuronal vulnerability by activating pro-apoptotic and pro-inflammatory pathways, which may contribute to both acute and chronic neurological impairments.

While this study provides valuable insights into the molecular mechanisms underlying a hypoxia-induced brain injury, several important questions remain. Future research should focus on exploring neuroprotective strategies, including both pharmacological and nutraceutical interventions aimed at mitigating oxidative stress, neuroinflammation, and apoptosis. Agents such as antioxidants, anti-inflammatory compounds, and modulators of cell death pathways hold promise for reducing hypoxia-related neural damage.

Additionally, longitudinal studies are needed to evaluate the long-term consequences of chronic hypoxic exposure and to determine the extent to which molecular and functional alterations are reversible upon reoxygenation. Such investigations will be crucial for understanding the persistence of hypoxia-induced brain changes and the brain’s potential for recovery.

Further research should also address the regional and cellular specificity of oxidative and inflammatory responses. In particular, elucidating the roles of discrete brain regions and the contributions of glial cells—such as microglia and astrocytes—will enhance our understanding of the cellular dynamics involved in hypoxic brain injury.

Given the increasing relevance of high-altitude exposure in contexts such as tourism, sports, and occupational activities, translational studies in human populations are essential. These efforts will help validate the clinical significance of experimental findings and support the development of individualized neuroprotective strategies.

Moreover, the integration of omics technologies—including transcriptomics, proteomics, and metabolomics—could provide a more comprehensive view of the molecular networks disrupted by hypoxia. These approaches may facilitate the identification of novel biomarkers and therapeutic targets, ultimately improving our capacity to predict, prevent, and treat high-altitude-induced brain injury.

In summary, addressing these research gaps will further elucidate the complex pathophysiological mechanisms of high-altitude hypoxia and support the advancement of effective preventive and therapeutic interventions.

## Data Availability

It can be requested from the authors.

## Abbreviations

A1	exposed to high altitude 3151 m
A2	exposed to high altitude 4214 m
ROS	reactive oxygen species
MDA	malondialdehyde
SOD	superoxide dismutase
GAPDH	glyceraldehyde-3-phosphate dehydrogenase
BCL2	B-cell lymphoma 2
CASP3	caspase 3
BAX	BCL2 associated X
BNIP3	BCL2 interacting protein 3
APAF-1	apoptotic protease activating factor 1
NRF2	nuclear erythroid 2- related factor 2
GPx	glutathione peroxidase
COX2	cyclooxygenase 2
TNF-α	tumor necrosis factor alpha
NF-kB	nuclear factor kappa B
